# The impact of COVID-19 on patients with chronic pain seeking care at a tertiary pain clinic

**DOI:** 10.1038/s41598-022-10431-5

**Published:** 2022-04-19

**Authors:** Maisa S. Ziadni, Dokyoung S. You, Eric M. Cramer, Steven R. Anderson, Gabrielle Hettie, Beth D. Darnall, Sean C. Mackey

**Affiliations:** Systems Neuroscience and Pain Lab, Division of Pain Medicine, Stanford University School of Medicine, 1070 Arastradero Road, Suite 200, Palo Alto, CA 94304 USA

**Keywords:** Quality of life, Viral infection

## Abstract

Empirical data on the health impacts of the COVID-19 pandemic remain scarce, especially among patients with chronic pain. We conducted a cross-sectional study matched by season to examine patient-reported health symptoms among patients with chronic pain pre- and post-COVID-19 pandemic onset. Survey responses were analyzed from 7535 patients during their initial visit at a tertiary pain clinic between April 2017–October 2020. Surveys included measures of pain and pain-related physical, emotional, and social function. The post-COVID-19 onset cohort included 1798 initial evaluations, and the control pre-COVID-19 cohort included 5737 initial evaluations. Patients were majority female, White/Caucasian, and middle-aged. The results indicated that pain ratings remained unchanged among patients after the pandemic onset. However, pain catastrophizing scores were elevated when COVID-19 cases peaked in July 2020. Pain interference, physical function, sleep impairment, and emotional support were improved in the post-COVID-19 cohort. Depression, anxiety, anger, and social isolation remained unchanged. Our findings provide evidence of encouraging resilience among patients seeking treatment for pain conditions in the face of the COVID-19 pandemic. However, our findings that pain catastrophizing increased when COVID-19 cases peaked in July 2020 suggests that future monitoring and consideration of the impacts of the pandemic on patients’ pain is warranted.

## Introduction

The COVID-19 pandemic has prompted healthcare organizations to make drastic changes in the delivery of pain care services to minimize virus exposure risks. For instance, elective medical procedures, in-person visits, and multidisciplinary services are being limited, while telehealth, digital treatment, and virtual reality expanded^1^. These rapid changes require healthcare clinicians and patients to adjust to many unknowns. Understanding the impact of the pandemic on the health status of patients seeking treatment for pain conditions is critical to help identify areas of growing need, evaluate any gaps in services, and ensure optimal pain care.

To date, empirical data on the health impacts of the pandemic are scarce. Some evidence suggests that humans are overall resilient and are adjusting well during the pandemic^2–4^, while others show that the pandemic has adversely impacted adults’ physical and mental health. Such adverse impacts include a significant reduction in daily physical activity^5^, increase in depression symptoms^6,7^, and more frequent alcohol use^8^. These adverse impacts are expected to be even more evident in medically vulnerable populations. A survey study on patients with cancer found a significant reduction in cognitive and social function during the COVID-19 pandemic^9^. Patients with chronic pain conditions, which may be triggered or exacerbated by psychosocial stressors^10,11^, are likely to be similarly vulnerable to the adverse impacts of the pandemic^12^. Two Pan-Canadian studies found that adults living with chronic pain reported increased pain, increased psychological distress and lack of access to treatment^13,14^. Similarly, a US-based study found increased pain intensity and interference among patients with chronic pain^15^, within a limited time-period following COVID-19 social distancing mandates.

A conceptual model has been proposed to explain how changes in social environment imposed by the COVID-19 pandemic influence the health status of patients with chronic pain^16^. In this model, the pandemic is conceptualized as a social threat. People living with chronic pain face challenges in maintaining optimal levels of social well-being even before the pandemic. Then, rules and systems to control the spread of COVID-19 such as lockdown and social distancing may worsen patients’ social isolation, increased child/family care burden, interpersonal conflict, access to pain care, and perceived injustice view of society and social inequality. Cumulative evidence suggests that these social threats worsen pain levels, pain-related symptoms, psychosocial function, and pain interference^17–22^. Evidence supporting this model was found in a cross-sectional online survey of community-dwelling adults with chronic pain who reported an increase in average pain intensity, pain flare-up episodes, distress, pain interference, and impaired sleep during the pandemic^23^.

The aim of the current study was to evaluate the physical and psychosocial health impacts of the COVID-19 pandemic on patients seeking treatment for chronic pain. A secondary aim is to identify potential sex differences in the pandemic’s health impact. Worse health status has been observed in female patients seeking treatment for chronic pain in primary care^24^ and pain clinic^25^ settings. Yet it is unknown whether the pandemic has a greater impact on female patients’ health status. We hypothesized that pain and pain-related symptoms, psychological distress, and social function would worsen after the COVID-19 pandemic onset. Second, we hypothesized that female patients would report greater negative health impacts of the pandemic compared to male patients.

## Methods

### Study setting and participants

**Figure 1:**
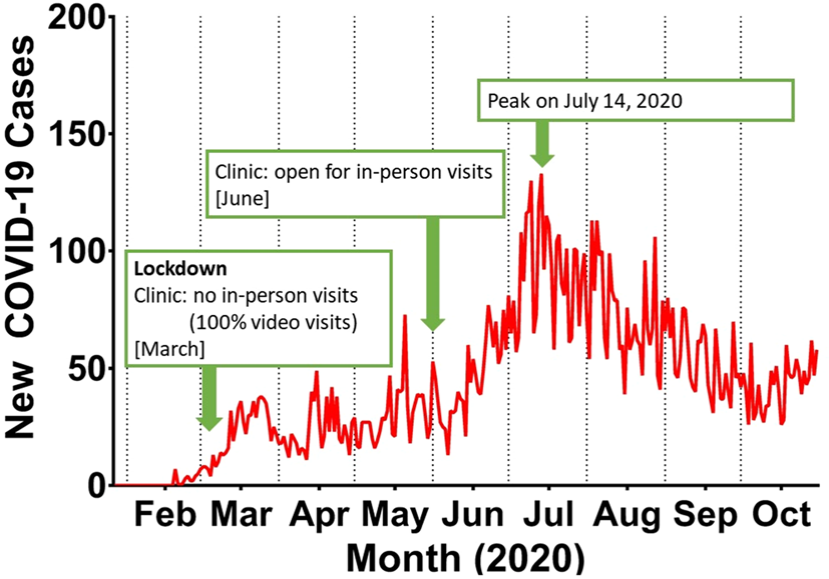
New COVID-19 cases in San Mateo county where the Stanford Pain Management. Center is located. Data were obtained from https://www.smchealth.org/coronavirus-health-data.

We conducted a cross-sectional study matched by season to examine patient-reported health symptoms among patients with chronic pain pre- and post-COVID-19 pandemic onset. Study procedures included a retrospective data analysis from patient surveys collected during initial visits to a tertiary pain clinic. Data was collected through CHOIR (Collaborative Health Outcomes Information Registry) to characterize patients’ physical, psychological, and social function during the months of pandemic lockdown, which went into effect in March of 2020 in the California Bay Area, and compared it to a control pre-COVID-19 time period. Our clinic offered virtual visits only in the first three months of the active lockdown (March–May) and re-opened for in-person visits in June 2020. See Fig. 1 for a timeline of COVID-19 cases in San Mateo County with clinic lockdown and opening dates indicated.

The current study included a total of 7535 patients at the Stanford Pain Management Center between 2017 and 2020. The post-COVID-19 onset cohort included 1798 patients who completed initial surveys collected between April–October 2020, and the control pre-COVID-19 cohort included 5737 patients who completed initial surveys collected between April–October 2017, 2018, and 2019. To control for seasonal effects, we constrained our control cohort to the same months as the post-COVID-19 onset cohort for the preceding 3 years. Stanford University’s Institutional Review Board (IRB) approved the study procedures. Informed consent was waived by the IRB, as CHOIR data were collected for clinical care and quality improvement purposes. The research was conducted in full compliance with the APA Ethical Principles in the treatment of human participants.

### Measures

#### Demographic

Patient sex, race/ethnicity, disability status, employment status, marital status, and age were collected to assess demographic characteristics. We also included in analyses the number of doctor’s office and ER visits over the past six months and the number of painful sites using a body map^26^. Finally, we analyzed current drinking status and number of drinks per week for those endorsing alcohol use.

#### Pain intensity

Pain intensity was assessed on a numerical rating scale (NRS) using the PROMIS Pain Intensity scale^27^. Respondents were asked to rate their average pain intensity over the previous 7 days on a scale of 0–10. Assessment of pain intensity using an NRS has been supported in prior studies^28^.

#### Pain catastrophizing

The Pain Catastrophizing Scale (PCS) is a 13-item questionnaire that assesses distress regarding the cognition and emotion associated with actual or anticipated pain^29^. The PCS comprises three different subscales: rumination, magnification, and helplessness. Patients rate each item on a 5-point Likert Scale (0 = Not at all to 4 = All the time). The PCS has a minimum score of 0 and a maximum score of 52 with higher scores indicative of more catastrophic thinking.

#### The patient-reported outcomes measurement information system (PROMIS)

The PROMIS measures are well-validated and widely used to assess physical and psychosocial health status in patients with chronic illnesses, including chronic pain^30–35^. Detailed information about the measure development and validation is available at http://www.healthmeasures.net. All PROMIS measures in the current study were administered using computerized adaptive testing (CAT). T-scores were calculated for each patient (*M* = 50, *SD* = 10). PROMIS CAT instruments were administered for pain interference, physical function, fatigue, sleep impairment, depression, anxiety, anger, social isolation, emotional support, and satisfaction with roles and activities. Higher scores on each PROMIS measure generally indicate greater severity of each symptom domain. However, higher scores on PROMIS physical function, emotional support, and satisfaction with roles and activities indicate better health status in each domain.

### Statistical analysis

Data were checked for completeness and nonsensical input (e.g., negative ages) before each demographic variable was quantified. We analyzed data of people who completed all PROMIS measures and the PCS. Subsequently, missing values existed only in demographic data. The proportion of patients belonging to each demographic variable were compared between pre- and post-COVID-19 pandemic onset cohorts using chi-squared tests to detect significant changes in cohort composition across timepoints. Additionally, age and the number of office visits, ER visits, painful sites, and weekly drinks were compared between the pre- and post-COVID-19 cohorts using a two-samples t-test.

SPSS 26 was used to conduct 2 (sex: female and male patients) by 2 (time: pre- and post-COVID-19 pandemic onset) by 7 (month: April to October) weighted-means MANOVAs for three sets of variables: (a) pain rating and PCS scores, (b) PROMIS physical health measures, and (c) PROMIS psychosocial health measures. Due to unbalanced samples, with small sample sizes for the post-COVID-19 cohorts, Type 1 (sequential) Sum of Squares was used for MANOVA and post-hoc univariate analyses^36^. To reduce Type I error for multiple comparisons, *p* values from post-hoc analyses were false discovery rate (FDR) adjusted in R using the Benjamini and Hochberg (BH) procedure^37^. Addressing our primary research aim, which was to investigate impacts of the COVID-19 pandemic on patients’ health status, we examined the main effect of time and its interaction with month predicting patient health status. To address our secondary research aim, which was to examine sex differences in the effect of the pandemic, we focused on the main effect of time and its interaction with sex and/or month.

## Results

### Sample characteristics

Characteristics of both cohorts are presented in Table 1. The majority of the patients across post-COVID-19 pandemic onset and pre-COVID-19 cohorts were female (67.3 and 71.0%), White/Caucasian (58.4 and 60.2%), and middle-aged [*M* = 51.4 (17.0) and *M* = 50.2 (17.5)], respectively. Most patients were not on disability across both cohorts (78.8% and 79.9%), and over half of patients reported that they were not currently working (55.7% and 56.8%). In sum, no significant differences were noted across cohorts on demographic variables except for sex and age, with the post-COVID-19 cohort including slightly more female patients and slightly younger in age.

**Table 1:** Sample characteristics of pre- and post-COVID-19 pandemic onset cohorts.

Characteristic	Pre-COVID-19 pandemic N = 5737		Post-COVID-19 pandemic onset N = 1798		*Χ*^2^	*p*
	n	(%)	n	(%)		
**Gender**					**8.57**	**0.003**
Female	3860	(67.3)	1276	(71.0)		
Male	1877	(32.7)	522	(29.0)		
**Race/ethnicity**					1.87	0.171
White	3348	(58.4)	1082	(60.2)		
Others^^^	2389	(41.6)	716	(39.8)		
(Asian)	569	(9.9)	187	(10.4)		
(Hispanic/Latino)	448	(7.8)	155	(8.6)		
(Black or African American)	278	(4.8)	55	(3.1)		
(bi-racial, Native Indians, etc.)	824	(14.4)	229	(12.7)		
(Missing/refused to disclose)	270	(4.7)	90	(5.0)		
**Disability status**					1.03	0.309
Not on disability	4521	(78.8)	1437	(79.9)		
On disability	1216	(21.2)	361	(20.1)		
**Employment status**					0.73	0.391
Currently working	2542	(44.3)	776	(43.2)		
Not currently working	3195	(55.7)	1022	(56.8)		
**Marital status**					2.77	0.598
Married	3137	(55.4)	972	(54.7)		
Never married	1144	(20.2)	380	(21.4)		
Divorced/separated	758	(13.4)	223	(12.6)		
Living together	345	(6.1)	119	(6.7)		
Widowed	277	(4.9)	82	(4.6)		
(missing)^^^	76	–	22	–		
**Drinking alcohol**					0.37	0.544
Yes	1864	(41.6)	648	(40.2)		
No	2621	(58.4)	963	(59.8)		
(missing)^^^	1252	–	187	–		

	*M*	(*SD*)	*M*	(*SD*)	*t*	*p*^*^*^
Age	**51.4**	**(17.0)**	**50.2**	**(17.5)**	**2.96**	**0.015**
Doctor’s office visits	7.0	(8.8)	6.6	(8.3)	2.04	0.068
ER visits	0.8	(2.4)	0.8	(2.5)	0.37	0.714
Painful sites (body map)	**11.5**	**(12.3)**	**12.3**	**(12.3)**	**− 2.50**	**0.030**
Number of drinks per week*	3.7	(4.5)	3.6	(6.8)	0.57	0.563

Bold font indicates significance at *p* < 0.05. Others^^^: Break downs of other races are listed below in parenthesis; *p*^*^*^ FDR adjusted p values; *n = 1709 of pre-covid-19 sample and n = 583 of post-covid-19 sample provided the information.

The proportion of patients drinking alcohol was similar between the two cohorts (41.6% and 40.2%, *p* = 0.544). Among patients drinking alcohol, the number of drinks per week were also not significantly different between the pre- and post-COVID-19 cohorts (3.7 and 3.5 drinks, *p* = 0.358). While no significant differences in healthcare utilization were found (doctor’s office and ER visits, *p*s > 0.068), the post-COVID-19 cohort endorsed significantly more painful sites on the body map (*M* = 12.3) than the pre-COVID-19 cohort (*M* = 11.5, *p* = 0.030, Table 1).

### Comparing pain ratings and pain catastrophizing scores between pre- and post-COVID-19 onset cohorts

**Figure 2:**
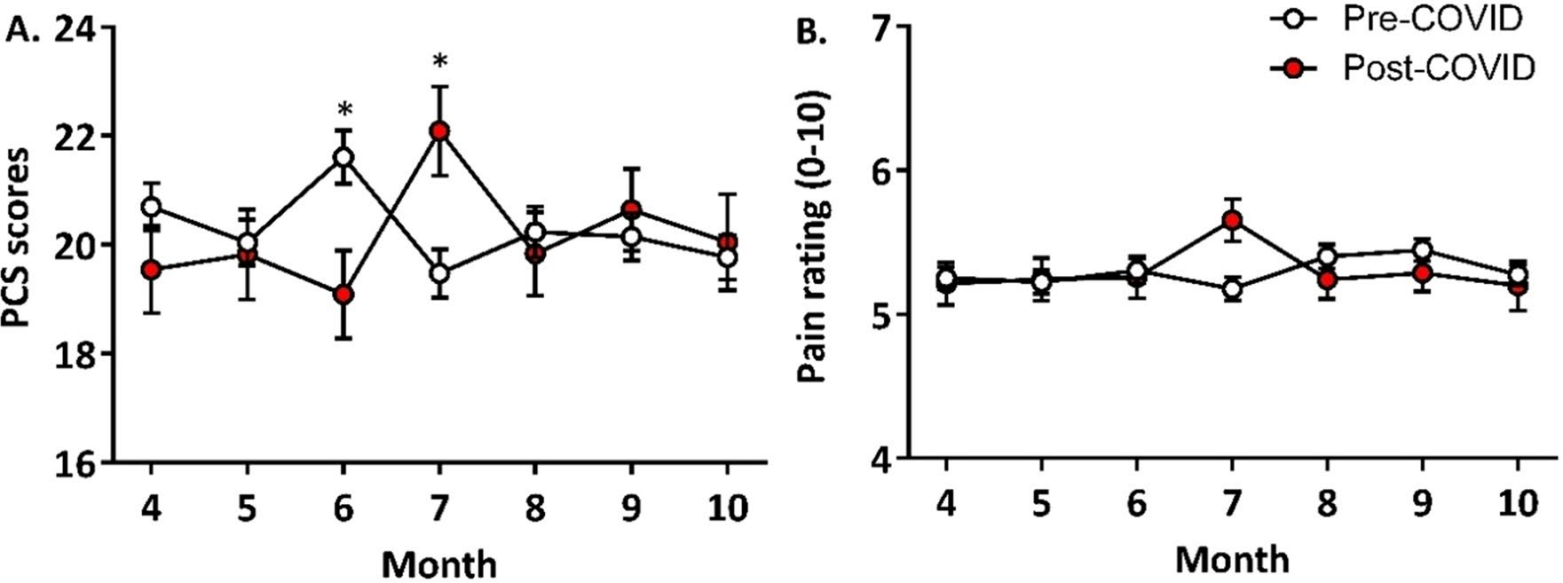
Comparing the changes in PCS scores (**A**) and pain ratings (**B**) by month between the pre- and post-COVID-19 cohorts.

Two (sex: male and female) by two (time: pre- and post-COVID-19 pandemic onset) by seven (month: April to October) weighted-means MANOVAs were conducted for pain ratings and pain catastrophizing scores (Table 2). There was a significant main effect of sex (*p* < 0.001) and a significant interaction between time and month (*p* = 0.020). Post-hoc univariate analyses showed that the time-by-month interaction was significant for PCS scores [*F*(6, 7,507) = 2.88, *p* = 0.016] but not for pain ratings (*p* = 0.143). Specifically, PCS scores significantly differed between cohorts in June [*F*(1, 938) = 7.09, *p* = 0.028] and July [*F*(1, 998) = 7.98, *p* = 0.028], but did not differ in the other months (*p*s > 0.478). In June, PCS scores were significantly lower in the post-COVID-19 cohort (*M* = 19.1, *SD* = 12.6) compared to the pre-COVID-19 cohort (*M* = 20.0, *SD* = 12.8, ƞ^2^ = 0.007; Fig. 2A). In July, the opposite pattern was observed: PCS scores were significantly higher in the post-COVD-19 cohort (*M* = 22.1, *SD* = 12.6) compared to the pre-COVID cohort (*M* = 19.5, *SD* = 12.3, ƞ^2^ = 0.008; Fig. 2A).

**Table 2:** Three-way MANOVA for pain ratings, PCS scores, and PROMIS measures.

Measures		Λ	*F*	*df*	*p*
Average pain intensity PCS scores	**Sex**	**0.994**	**22.62**	**(2, 7506)**	**< 0.000**
	Time	1.000	0.07	(2, 7506)	0.932
	Month	0.998	0.95	(12, 15,012)	0.455
	**Time × month**	**0.997**	**2.01**	**(12, 15,012)**	**0.020**
	Sex × time	0.999	2.34	(2, 7506)	0.096
	Sex × month	0.999	0.70	(12, 15,012)	0.752
	Sex × time × month	0.999	0.79	(12, 15,012)	0.660
Pain interference Physical function Fatigue Sleep impairment	**Sex**	**0.984**	**30.26**	**(4, 7504)**	**< 0.001**
	**Time**	**0.998**	**4.41**	**(4, 7504)**	**0.001**
	Month	0.996	1.24	(24, 26,180)	0.196
	Time × month	0.996	1.19	(24, 26,180)	0.240
	**Sex** × **time**	**0.999**	**2.74**	**(4, 7504)**	**0.027**
	Sex × month	0.997	1.01	(24, 26,180)	0.443
	Sex × time × month	0.998	0.67	(24, 26,180)	0.887
Depression Anxiety Anger Social isolation Emotional support Satisfaction with social roles and activities	**Sex**	**0.983**	**22.10**	**(6, 7502)**	** < 0.001**
	**Time**	**0.996**	**5.12**	**(6, 7502)**	** < 0.001**
	Month	0.995	1.04	(36, 32,946)	0.396
	Time × month	0.997	0.69	(36, 32,946)	0.917
	Sex × time	0.999	0.71	(6, 7502)	0.643
	Sex × month	0.994	1.25	(36, 32,946)	0.917
	Sex × time × month	0.997	0.71	(36, 32,946)	0.906

Bold font indicates significance at *p* < 0.05.

Taken together, we found small but significant differences in pain catastrophizing in June and July 2020 compared to pre-pandemic months. PCS scores were significantly lower after the onset of the pandemic in June 2020 when the clinic was re-opened for in-person visits, and increased in July 2020 when new COVID-19 cases peaked. However, these changes in PCS scores in June and July 2020 were not paralleled by changes in pain ratings (Fig. 2B).

### Comparing PROMIS-physical health measures between pre- and post-COVID-19 onset cohorts

**Figure 3:**
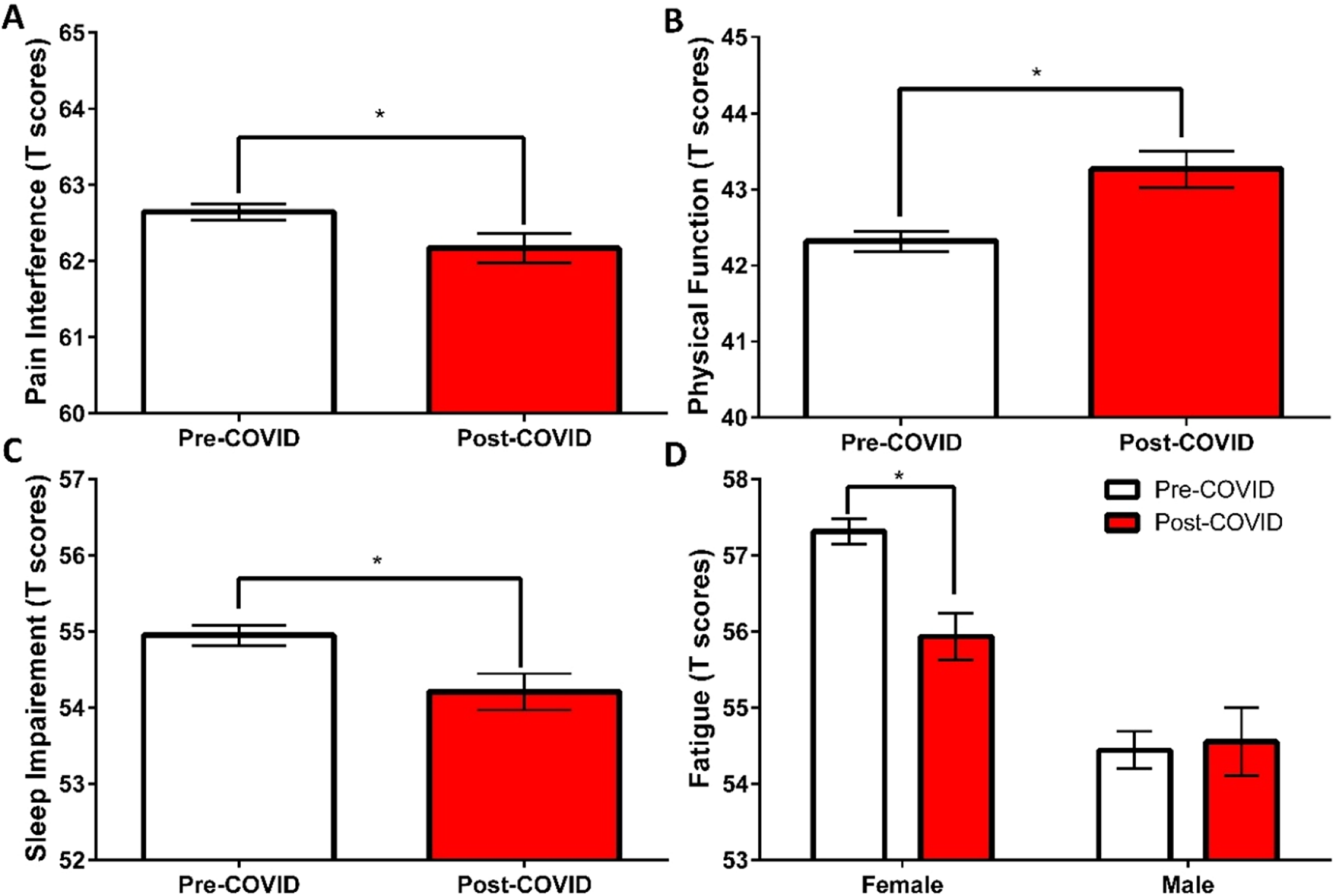
Comparing PROMIS-pain interference (**A**), physical function (**B**), sleep impairment (**C**), and fatigue (**D**) T-scores between pre- and post-COVID-19 pandemic onset cohorts.

Two (sex: male and female) by two (time: pre- and post-COVID-19 pandemic onset) by seven (month: April to October) weighted-means MANOVAs were conducted for PROMIS-pain interference, physical function, fatigue, and sleep impairment T-scores (Table 2). There were significant main effects of sex (*p* < 0.001) and time (*p* < 0.001), as well as a significant time-by-sex interaction (*p* = 0.027). Post-hoc univariate analyses revealed significant effects of time for the pain interference, physical function, fatigue, and sleep impairment scores, *F*s > 5.10, *p*s < 0.036, ƞ^2^ = 0.001, suggesting a slight improvement in these physical health measures (Fig. 3A–D). Post-hoc univariate analyses revealed a significant sex-by-time interaction only for fatigue, *F*(1, 7659) = 5.84, *p* = 0.027, ƞ^2^ = 0.001, but not for the other measures (*p*s > 0.338). PROMIS-fatigue T-scores were significantly lower in the post-COVID-19 cohort versus pre-COVID-19 cohort among female patients, *F*(1, 5134) = 16.66, *p* < 0.001, but not among male patients (*p* = 0.838, Supplementary Table).

Contrary to our primary hypothesis, we found small but significant improvements in pain interference, physical function, and sleep impairment in the post-COVID-19 cohort. Addressing our secondary hypothesis regarding sex differences, we found that female patients in the post-COVID-19 cohort showed significant improvements in fatigue symptoms (Fig. 3D).

### Comparing PROMIS-psychosocial health measures between pre- and post-COVID-19 onset cohorts

**Figure 4:**
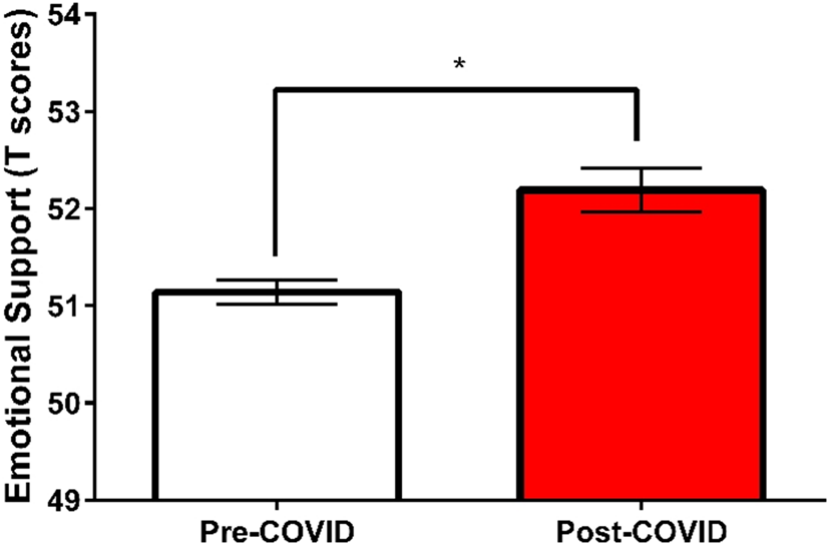
Comparing the PROMIS-emotional support T-scores between the pre- and post-COVID-19 cohorts.

Two (sex: male and female) by two (time: pre- and post-COVID-19 pandemic onset) by seven (month: April to October) weighted-means MANOVAs were conducted for PROMIS-depression, anxiety, anger, emotional support, social isolation, and satisfaction with social roles and activities (Table 2). There was a significant main effect of sex (*p* < 0.001) and time (*p* < 0.001). Post-hoc univariate analyses showed that the main effect of time was significant for emotional support *F*(1, 7,553) = 15.01, *p* < 0.001, ƞ^2^ = 0.002, but not for the other measures (*p*s > 0.084). PROMIS-emotional support T-scores were significantly higher in the post-COVID-19 cohort (*M* = 52.2, *SD* = 9.5) than the pre-COVID-19 cohort (*M* = 51.2, *SD* = 9.5, Fig. 4).

These results suggest that patients in the post-COVID-19 cohort reported small but significant improvements in emotional support compared to the pre-COVID-19 cohort. Contrary to our hypothesis, we did not see a significant worsening of depression, anxiety, anger, social isolation, or satisfaction with social roles and activities in the post-COVID-19 patient cohort.

## Discussion

We conducted a cross-sectional study matched by season to examine patient-reported health symptoms in patients with chronic pain pre- and post-COVID-19 pandemic onset (April–October 2020). To our knowledge, this is the first study to investigate the impacts of the COVID-19 pandemic and sex on the health status of patients with chronic pain seeking treatment.

Drastic changes in social interactions and medical practice imposed by the COVID-19 pandemic have created unique challenges to chronic pain management. Already, there is emerging evidence in the literature of the negative impacts of the pandemic on patients with chronic pain. Community-dwelling adults with chronic pain in one study reported worse pain and health status compared to their recall of pre-pandemic health status^23^, and pain physicians have reported observations of increased pain levels in patients visiting their clinics after the pandemic onset compared to their recall of typical patients prior to the pandemic^38^. Given these findings, in the present study we hypothesized that the onset of the COVID-19 pandemic would be associated with worse pain and physical and psychosocial health among patients with chronic pain visiting our clinic. Consistent with our hypothesis, we found that patients’ pain catastrophizing was significantly lower after the onset of the pandemic in June 2020 (when the clinic was re-opened for in-person visits), and higher in July 2020 (when new COVID-19 cases peaked in Santa Clara and San Mateo County) compared to pre-pandemic months. Counter to our hypothesis, however, we did not observe a similar change in pain ratings, and in fact observed small but significant improvements in patients’ pain interference, physical function, and sleep impairment in the post-COVID-19 sample. These findings were not consistent with those reported among individuals living with chronic pain in two Pan-Canadian studies^13,14^. Similarly, addressing our secondary research aim of investigating sex differences in the impact of the pandemic, we found that female patients in the post-COVID-19 cohort showed significant improvements in fatigue symptoms.

Overall, we found that the pandemic did not adversely influence markers of physical and mental health among patients seeking treatment for pain in our clinic. Our post-COVID-19 cohort also reported small but significant increases in emotional support and no significant changes in social isolation and satisfaction with social roles. Although this is contrary to published findings to date^13,14^, an important distinction is that the current study sample were in treatment at the Stanford Pain Center and continued to receive virtual treatment during the pandemic. Treatment was continuously available via virtual-platforms throughout the study timeframe, and engagement was very high. In addition, patients reported access to increases in emotional support in the post COVID group. These factors likely buffered the impacts of COVID-19 and were key contributors to the observed improvements. In addition, study findings are consistent with prior findings of resilience in response to the pandemic^2–4^, which may have buffered the effects of the pandemic on stress and emotional functioning. Furthermore, small but significant changes in pain interference, sleep impairment, and physical function were all in the direction of improvement after the pandemic. These results should also be considered in the context of our patients’ social situations. Most of our patients were married or living together, of which proportions were not significantly different between the pre- (68.8%) and post-COVID-19 cohorts (69.3%). Additionally, employment status, disability status, and the numbers of doctor’s office visits and ER visits for the past six months were not significantly different between the pre- and post-pandemic cohorts.

Past research has suggested worsening outcomes following epidemics and past natural disasters^39–41^. Alcohol use, PTSD, anxiety, anger, fear of contagion, perceived risk, and distrust emerge as potential effects from the COVID-19 pandemic^40^. As such, future work should focus on identifying risk and protective factors that may mitigate or exacerbate negative impacts, including mental health status, substance use, and related demographic variables. Importantly, current study findings support the notion that having access to treatment in the context of a pandemic was likely protective and allowed patients to benefit from the care in spite of the pandemic. Additionally, research on resilience among individuals with chronic pain is growing^42^, and studies show that certain positive characteristics, such as optimism, pain acceptance, and purpose in life have been associated with increased resilience to chronic pain^43^. It is possible that the development of coping strategies required to manage chronic pain may be protective when facing a widespread disruptive event such as a pandemic.

However, it is possible that it is still too early to assess the full impacts of the pandemic on limiting access to care, medications, or other critical services. With a pattern of cyclic surges of COVID-19 cases and new strains emerging, patients with chronic pain will continue for the foreseeable future to experience social and emotional challenges arising from the pandemic. In our clinic, when new COVID-19 cases peaked in July 2020, our patients’ pain catastrophizing scores increased compared to the same month of the previous three years. The extended social and emotional toll of the pandemic, with multiple surges of new COVID-19 cases and associated lockdowns, may be increasing patients’ catastrophic thinking about their pain. Pain catastrophizing is a negative cognitive and emotional response pattern to actual or anticipated pain^44,45^ that has been previously linked to increased pain report, opioid use, and pain-related disability^46–48^. More study is warranted to understand the long-term physical and mental health impacts of the pandemic. Alternatively, the Substance Abuse and Mental Health Services Administration (SAMHSA) acknowledges that lack of health insurance and limited access to mental and behavioral health services are a major health disparity issue during the COVID-19 pandemic^49^. Yet, this is not the case for the majority of the patients seeking treatment at our pain clinic. Future study in more vulnerable patient populations, who may lack health insurance or have substantial barriers to care, is needed. Even though the confounding impact of alcohol use was examined in this study, future work should assess the confounding effect of other and recreational substance use, in addition to mental health status, on patient-outcomes.

Several limitations of the current study are worth noting. First, the cross-sectional design of our study precludes making causal inferences. The negative effects of the pandemic may increase over time with persistent virus spread, worsening economic hardship, social isolation, and inability to obtain medications. Hence, future longitudinal studies are required to assess changes in pain and functioning over time. Next, selection bias is another potential limitation, as it is possible that the patients most severely impacted by the COVID-19 pandemic were not sampled in our study because they were not able to successfully obtain care at our clinic. Finally, our study cohorts were recruited from a pain management clinic located in the San Francisco Bay Area. Regional variability in COVID-19 caseloads, mortality, and government-mandated lockdowns may influence the negative impacts of the pandemic on patients with pain. In San Mateo County, where the Stanford Pain Management Center is located, there have been considerably fewer deaths due to COVID-19 than in many other parts of the United States^50^. Increased severity of an outbreak in a locality may result in increased detrimental physical and psychosocial impacts in patients with pain and other chronic illnesses. Notably, the current study was not designed to specifically query COVID-19 impacts. Future research is needed to probe patient experiences specific to the COVID-19 pandemic, such as testing positive for COVID-19, experiencing long-term effects of infection, loss of employment and related financial impacts, barriers to accessing healthcare, and childcare burden, and whether these new stressors correlate with pain-related outcomes.

Study findings are encouraging and could be bolstered by considering adjunctive digital health solutions to further address COVID-19-related misinformation, provide support, and maintain continuity of care^51^. For instance, we showed that a zoom-delivered behavioral health intervention “Empowered Relief” imparts enduring benefits^52^; this and other online-delivered interventions, as well as on-demand digital treatments such as virtual reality^53^, may be integrated into pain care pathways broadly. In light of the urgent clinical need and patient preference for digital services due to the COVID-19 pandemic, future studies could focus on assessing the impact of online-health communities, using phone calls, and AI-based approaches for providing counseling and promoting self-management skills among patients with chronic pain^51^.

## Conclusion

Despite these limitations, this is the first study to report data on patients with chronic pain, controlling for seasonal effects, and examining sex differences in potential adverse health impacts of the pandemic. A notable strength of this study is the multidimensional nature of the data, which included both psychosocial and pain-related measures. Finally, we used a learning health system to examine changes in physical and psychosocial health among a large number of real-world patients seeking treatment for pain before and after the pandemic onset. Subsequently, our results were free of recall bias and ecologically valid in understanding the health status of patients with whom treating physicians would interact in their daily practice. In conclusion, our findings overall provide evidence of encouraging resilience among patients seeking treatment for pain conditions in the face of the COVID-19 pandemic. However, our finding that patients’ pain catastrophizing increased when COVID-19 cases peaked points to the need for future study and monitoring of patients as the effects of the pandemic continue.

## Supplementary Information

Supplementary Table 1.

## Data Availability

The datasets generated during and/or analyzed during the current study are available from the corresponding author on reasonable request.
