# Supplementary material for: The impact of COVID-19 on patients with chronic pain seeking care at a tertiary pain clinic
Source: Sci Rep. 2022 Apr 19;12:6435. doi: 10.1038/s41598-022-10431-5 (PMC9017421; doi:10.1038/s41598-022-10431-5)
Supplement: Supplementary file 1 — Supplementary Table 1. [file 41598_2022_10431_MOESM1_ESM.docx]

Supplemental Table. Overall male and female patient health status post-COVID-19 pandemic onset in comparison to pre-COVID-19 pandemic.

|  | **Pre-COVID-19 Cohort** | | | | **Post-COVID-19 Cohort** | | | |
| --- | --- | --- | --- | --- | --- | --- | --- | --- |
|  | Female  *n* = 3,860 | | Male  *n* = 1,877 | | Female  *n* = 1,276 | | Male  *n* = 522 | |
| PROMIS | ***M*** | **(*SD*)** | ***M*** | **(*SD*)** | ***M*** | **(*SD*)** | ***M*** | **(*SD*)** |
| Average Pain | 5.4 | (2.3) | 5.0 | (2.4) | 5.4 | (2.3) | 5.1 | (2.3) |
| Pain Interference | 62.8 | (7.9) | 62.3 | (8.1) | 62.2 | (8.4) | 62.0 | (8.0) |
| Physical Function* | 42.0 | (10.0) | 42.9 | (10.2) | 42.9 | (10.3) | 44.2 | (10.1) |
| **Fatigue** | **57.3** | **(10.3)** | **54.4** | **(10.7)** | **55.9** | **(10.9)** | **54.5** | **(10.2)** |
| Sleep Impairment | 55.4 | (9.9) | 54.1 | (10.5) | 54.4 | (10.4) | 53.7 | (9.6) |
| Depression | 52.8 | (9.8) | 51.5 | (10.3) | 52.5 | (9.6) | 52.3 | (9.5) |
| Anxiety | 54.1 | (9.7) | 52.5 | (10.3) | 54.1 | (9.5) | 53.2 | (9.7) |
| Anger | 48.2 | (10.3) | 47.9 | (10.9) | 48.1 | (10.3) | 48.0 | (10.3) |
| Social Isolation | 47.0 | (9.4) | 46.5 | (9.9) | 46.7 | (9.3) | 46.8 | (9.4) |
| Emotional Support* | 51.6 | (9.3) | 5[8]0.2 | (9.9) | 52.7 | (9.3) | 50.9 | (9.9) |
| Satisfaction with  Roles and Activities* | 43.9 | (10.2) | 43.7 | (9.9) | 43.5 | (10.1) | 42.6 | (9.3) |
| Pain Catastrophizing | 20.6 | (12.6) | 19.6 | (12.5) | 20.0 | (13.0) | 20.6 | (12.7) |

*Note.* *Higher scores indicate better functioning on these indices of function
